# Serum Cortisol and Cardiovascular Disease Risk - A Potential Biomarker

**DOI:** 10.2174/011573403X328499241106064553

**Published:** 2025-01-01

**Authors:** Wei Jet Oo, Chooi Ling Lim, Mun Hon Goh, Rhun Yian Koh

**Affiliations:** 1 Division of Applied Biomedical Science and Biotechnology, School of Health Sciences, IMU University, 126, Jalan Jalil Perkasa 19, Bukit Jalil, 57000, Kuala Lumpur, Malaysia;; 2 Laboratory and Blood Services Department, National Heart Institute, 145, Jalan Tun Razak, 50400, Kuala Lumpur, Malaysia

**Keywords:** Serum cortisol, cardiovascular, stress, hormone, biomarker, mechanistic parameters

## Abstract

Cardiovascular disease (CVD), the leading cause of death globally, poses a significant burden on the healthcare sector. Its association with stress and Cushing’s Syndrome has driven cortisol, the ‘stress hormone,’ to be a potential candidate in determining CVD risk. Cortisol synthesis and release through the hypothalamic-pituitary-adrenal (HPA) axis are regulated by several hormones and receptors involved in the pathological cascade towards CVD. Evidence suggests that metabolic syndrome plays a major role in cortisol-mediated CVD risk. On the other hand, non-metabolic features are also implicated when the association between cortisol and CVD risk remains significant upon normalisation of metabolic parameters. Correspondingly, the treatment for hypercortisolism is often found effective in lowering CVD risk. Despite available evidence, several factors continue to hinder the clinical use of cortisol as a risk biomarker for CVD. This review provides an insight into the role of serum cortisol in CVD progression and risk, with emphasis on the mechanistic features and parameters.

## 1 INTRODUCTION

In 2019, more than a quarter of total global deaths (around 17.9 million people) can be attributed to cardiovascular disease (CVD). According to the American Heart Association, CVD-related deaths have risen by one million in solely one year. CVD, as a leading cause of death, also prevails in Malaysia, with coronary heart disease (CHD) and stroke ranking first and third, respectively. Owing to its chronic nature, CVD prevalence remains staggering, with a total of 244.1 million living cases of CHD and an approximated 89.1 million with stroke co-morbidity [1, 2]. Evidently, the global burden of CVD is significantly on the rise, thus revealing an urgent need for diagnostic and therapeutic breakthroughs.

Cortisol, typically known as the ‘stress hormone,’ is the predominant glucocorticoid (GC) produced by the adrenal cortex, which acts on cells with GC receptors [3, 4]. Apart from responding to stress, the diurnal circadian rhythm of cortisol secretion is also worth noting. Prominent functions of cortisol include immunoregulation by exerting anti-inflammatory actions and suppressive effects, energy mobilisation through glucose and protein metabolism, mediation of stress response, and vascular modification [3, 5]. Currently, the potential of serum cortisol as a risk biomarker for CVD is based upon their intrinsic relationship, whereby CVD often emerges as complication of Cushing’s Syndrome (CS) characterised by hypercortisolism. Meanwhile, studies show a persistently higher-than-normal CVD risk following resolved hypercortisolaemia [6, 7]. Hence, this review sheds light on the reliability of serum cortisol as a CVD risk biomarker and the uncertainties encountered in hopes of identifying new insights and potential gaps.

## 2 HPA AXIS AND SERUM CORTISOL

The hypothalamic-pituitary-adrenal (HPA) axis, through which cortisol synthesis and release occur, plays a major role in regulating cortisol levels. Cortisol release is initiated by hypothalamic activation upon awakening and encountering stress. Release of corticotropin-releasing hormone (CRH) and arginine vasopressin (AVP) follow to stimulate secretion of adrenocorticotropic hormone (ACTH) by the anterior pituitary gland, which subsequently induces the release of cortisol from the zona fasciculata of the adrenal cortex. While serving as fuel to the body, cortisol is also self-involved in its negative feedback inhibition by regulating the secretion of the 3 upstream hormones. Besides, GC receptors [GR] and mineralocorticoid receptors (MR) are also part of the negative feedback loop due to their different binding affinities for cortisol [8].

Once released, cortisol is either bound to cortisol-binding globulin (CBG) or freely circulating, and only the free form is physiologically active. Serum cortisol levels have been utilised as a diagnostic tool for cortisol-related diseases, either CS or Addison disease, also known as hyper- and hypocortisolism, respectively [3]. CVD, a gradually progressive chronic condition, challenges the immune system and induces stress-related cortisol levels. A similar pattern of cortisol elevation is seen in dengue patients, albeit at a more rapid pace, which highlighted the prospect of a cortisol biomarker for severe infections [9]. Therefore, it would make sense for clinicians to recommend further diagnostic investigations for CVD based on cortisol levels as a predictive tool. Additionally, it is important to consider the diurnal rhythm of cortisol secretion when evaluating CVD risk. To be more specific, the cortisol awakening response (CAR), which refers to its peak level 30 minutes post-awakening, should be included as one of the parameters for an accurate CVD risk assessment [10].

## 3 THE RELATIONSHIP BETWEEN CVD AND STRESS

Despite ongoing research, underlying mechanisms that link CVD and stress remain complicated. Results from meta-analyses indicate that among individuals who are socially isolated and under occupational stress, there is a respective 1.5- and 1.4-fold greater risk for CVD. Additionally, psychological stress, accompanied by emotions such as depression and anger, was found to increase the risks of acute cardiac events by 2.5 times [11]. Recent findings, which indicate a higher prevalence of early CVD onset among individuals with stress-related disorders, independent of family and medical history, further consolidate findings from previous studies [12]. Additionally, the prognosis for CVD patients can worsen as psychological stress elevates the risk of all-cause mortality by 51%. Though ample evidence has identified the relationship between CVD and stress, the underlying pathophysiology remains unclear. Bidirectionality has been suggested in this relationship where stress and CVD major risk factors have an influence on each other. Meanwhile, some found that even after adjustment for major risk factors, CVD risk can remain in the presence of stress [13-15].

HPA stress response involving cortisol is found to fluctuate with stressors’ duration and intensity. Chronic stress, which is cumulative in nature, is capable of impairing the HPA feedback mechanism by rendering the axis less flexible [16]. Besides, GR resistance that results in inflammation and enlargement of adrenal glands occurs, both of which are found to be associated with higher cortisol levels [17, 18]. In animal studies, consistent findings of chronic stress-enhancing ACTH response by inducing adrenal hyperplasia and hypertrophy, mainly in the zona fasciculata region, were obtained [19]. Neuroimaging studies further found that heightened amygdala activity upon stress can contribute to CVD risk by increasing atherosclerotic inflammation through enhanced hematopoiesis [20]. In the context of cortisol, an impaired feedback mechanism that results in a more sensitised HPA axis with the downregulation of GR and CBG can elevate cortisol levels [18]. Structural and functional cardiac abnormalities, not induced by hypertension *per se*, have also been described in CS patients. A study including both normotensive and hypertensive CS subjects showed a left ventricular (LV) mass index of 44.6 ± 14.7 g/m^2.7^ in comparison to 36.9 ± 10.1 g/m^2.7^ in controls after an adjustment of height to 2.7 m for weight correction [21]. In addition to increased LV mass index, lower LV, right ventricular, and right atrial ejection fractions are found reversible upon treatment for hypercortisolism, all of which showed no significant correlation with BP [22]. This demonstrates how intrinsic CVD and cortisol can be linked.

## 4 CORTISOL ALTERS METABOLIC FEATURES, LEADING TO CVD PROGRESSION

**Fig. (1):**
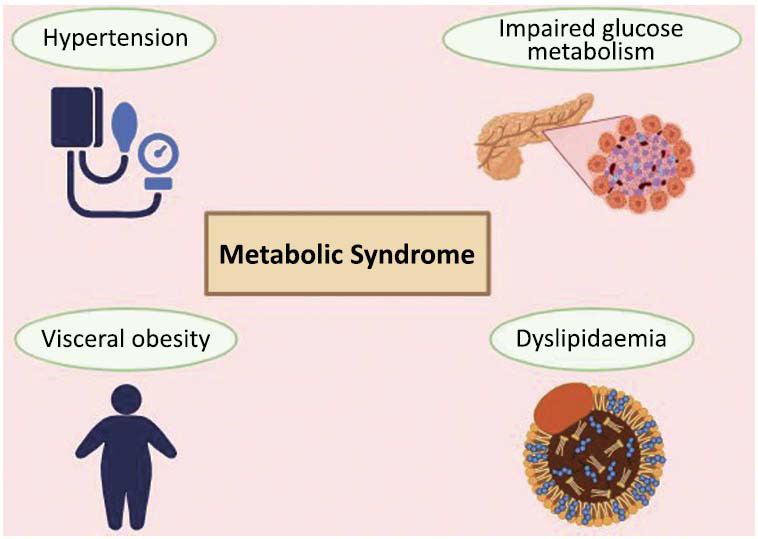
Metabolic syndrome consists of hypertension, impaired glucose metabolism, visceral obesity and dyslipidaemia. All of which are closely associated with CVD risk and can be mediated by cortisol. Created in BioRender. https://BioRender.com/v37t959.

Cortisol can heighten CVD risk by altering metabolic features, leading to hypertension, obesity, impaired glucose metabolism, and dyslipidaemia (Fig. **1**). These metabolic features, collectively known as the metabolic syndrome, are well-established CVD risk factors due to their role in endothelial dysfunction and plaque formation [23]. A study showed that among metabolism-related CVD deaths, half of them could have been preventable upon elimination of these metabolic risk factors [24]. Aside from exerting metabolic effects, the hemodynamic system is also under the influence of cortisol.

## 5 HYPERTENSION

Hypertension is a risk factor that exhibits the strongest evidence-based association with CVD [24-26]. In a study involving more than 500,000 participants, hypertension was found to account for the largest fraction following smoking in preventing cardiovascular mortality [24]. With no significant gender difference identified, hypertension presents as a characteristic feature of GC excess, which occurs in 25 – 93% of CS patients, and the incidence increases in individuals taking oral GC as a long-term effect [27-29]. Cortisol level following 1mg-dexamethasone suppression test (DST) was higher in hypertensive patients with non-functioning adrenal incidentaloma (NFAI) compared to normotensive NFAI patients, highlighting the role of cortisol in hypertension at levels lower than previously suggested [30]. A recent study conducted among systemic lupus erythematosus patients treated with GC further supports this by demonstrating reduced systolic and diastolic BP following GC tapering over time (baseline ≥10.0 mg/day to ≤7.5 mg/day) [31].

**Fig. (2):**
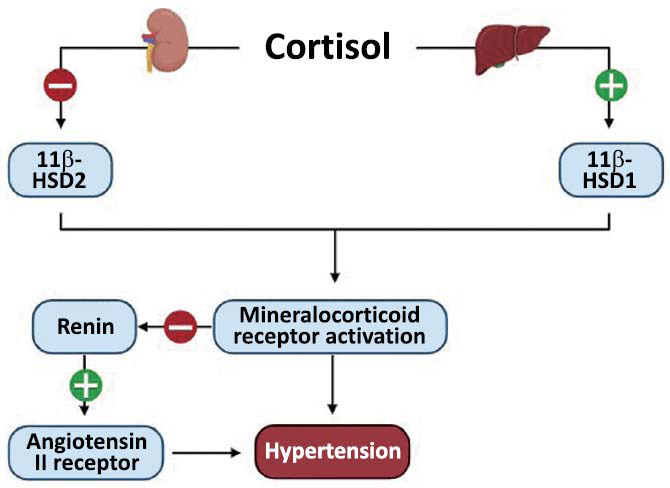
Mechanisms of cortisol leading to hypertension to increase CVD risk. 11β-HSD1 converts cortisol to its active form, whereas 11β-HSD2 exerts an opposing function. When 11β-HSD1 and 11β-HSD2 are upregulated and downregulated, respectively, activation of MR receptors occurs, increasing blood pressure at the renal and vascular levels. Created in BioRender. https://BioRender.com/l26i141.

Several mechanisms have been implicated in increasing CVD risk (Fig. **2**). Prominent ones include the roles of 11β-hydroxysteroid dehydrogenases [11β-HSDs] and angiotensin II. 11β-HSDs, consisting of type 1 (11β-HSD1) and 2 (11β-HSD2), are responsible for the interconversion between active cortisol and inactive cortisone. 11β-HSD1 which favours active cortisol, is involved in hypertension in an adipose-specific manner [32, 33]. This has been indicated by evidence of protective effect following adipose-specific 11β-HSD1 knockout in mice against metabolic effects arising from corticosterone excess, including hypertension [34]. One study further demonstrates its role in MR activation, where spironolactone, an MR blockade, reverses endothelial dysfunction by inhibiting 11β-HSD1 expression in perivascular fat without lowering GC levels [35].

Meanwhile, an excess of cortisol results in the under-expression of 11β-HSD2. With the same binding affinity to MR as aldosterone, cortisol elevates BP at the renal level by enhancing sodium reabsorption and potassium excretion. Another postulated mechanism is that 11β-HSD2 can cause salt-sensitive hypertension, which is more evident in mice models rather than in humans, thus requiring further investigation [36].

To compensate for renal changes induced by the MR-cortisol binding activity, suppression of renin level occurs. Instead of angiotensin II level being elevated, its action is enhanced through the upregulation of angiotensin II receptors, elevating BP at both renal and vascular levels [37, 38]. This strong association between hypercortisolism and hypertension is further supported by the treatment algorithm for hypertensive CS patients, which targets pathways interfered by glucocorticoid excess. Numerous clinical trials have validated the effectiveness of controlling BP by resolving hypercortisolaemia. The drug examples include pasireotide and mifepristone, as well as an ACTH inhibitor and a GR antagonist [39].

## 6 IMPAIRED GLUCOSE METABOLISM (IGM)

A meta-analysis comprising studies from a 10-year span (2007 - 2017) showed a 32% CVD prevalence in patients with type 2 diabetes mellitus (T2DM), whereas a 2 - 3-fold greater incidence was found in T2DM patients worldwide [40, 41]. The mechanism of IGM contributing to CVD risk can be best demonstrated through insulin resistance (IR), in which its progression to hyperglycaemia and dyslipidaemia will eventually lead to an inflammatory cascade for atherosclerosis development [42].

**Fig. (3):**
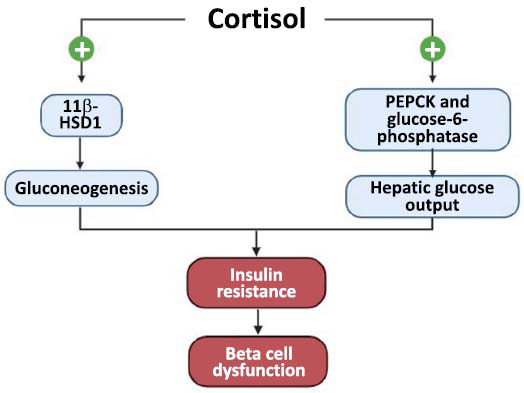
Cortisol induces IGM by causing insulin resistance and diabetes at a later stage. Through the respective enzymes, cortisol first leads to increased glucose synthesis and output, both of which are followed by insulin resistance. Insulin hypersecretion eventually results in a failure of beta cells, where insulin secretion declines and glucose levels persist. Created in BioRender. https://BioRender.com/k28n836.

Studies over the years indicate that 70% of endogenous CS patients suffer from IGM, whereas for individuals undergoing GC treatment, the risk of developing diabetes mellitus is found to be greater. A significant association with adrenal incidentaloma (AI) involving subclinical CS was also found, where insulin sensitivity is negatively correlated with serum cortisol after 1-mg DST (r-value = -0.65) while positively associated with serum ACTH (r-value = 0.62) [43, 44]. A prospective cohort study spanning a decade concluded that an elevated evening cortisol level may predict glucose metabolism impairment (Fig. **3**) [45].

Hypercortisolism-induced hyperglycaemia mainly occurs in the liver through the upregulation of enzymes involved in gluconeogenesis and disrupted inhibition of hepatic glucose output [43]. A recent study conducted among American Africans consolidates the relationship between cortisol and IGM, where a higher morning serum cortisol (MSC) is associated with higher fasting plasma glucose in both non-T2DM and T2DM individuals, additionally exacerbating IR in T2DM individuals. Despite considering racial factors, higher odds of T2DM prevalence have been determined with higher MSC [46]. IGM mediated by both MR and GR has also been proposed by studies on mice models. MR blockade increases adiponectin expression, which is found to be lower among IR and T2DM patients, while knockout of adipose-specific GR was followed by improved insulin sensitivity [47, 48]. Another study in the Japanese population showed a negative correlation between serum cortisol and beta cell function that persisted even after adjustment for IR [49]. Therefore, it can be deduced that cortisol impairs glucose metabolism not only by inducing IR but also by decreasing insulin secretion, which eventually results in diabetes.

## 7 ADIPOSITY

Visceral obesity, also known as abdominal obesity, is the most prevalent clinical feature of CS, accounting for 25 - 100% of cases [27]. This increased ectopic fat deposition around vital organs, regardless of hypercortisolism severity and period, has been extensively linked to CVD complications. Compared to body mass index (BMI), the accuracy of waist circumference (WC) in measuring visceral obesity has been proved higher in a study where WC in CS patients is higher than in controls with matching BMI [50, 51]. Another form of fat distribution is subcutaneous obesity, which is an excessive deposition of fat tissues underneath the skin, also associated with CVD risk. Generally, obesity is more common among GC users, where a two-fold difference in the use of exogenous GC within the last 3 months was found between obese (27%) and non-obese groups (13%) [52].

Adiposity as a major risk factor for CVD has been well-established, wherein now, cohort studies have found that visceral instead of subcutaneous obesity exhibits a stronger association with CVD. The influence of visceral obesity on CVD risk remains even after BMI adjustment, further supporting the aforementioned statement that BMI is a less sensitive marker for visceral obesity [53-55]. Another study that identified a higher risk for atrial fibrillation following higher cortisol levels also found that with adjustment of WC, lowering of risk is achievable [56]. Based on a recent study, the strong association between individuals with visceral obesity and adipokine dysfunction predisposes them to metabolic conditions like hypertension, IGM and dyslipidemia [57, 58].

Despite visceral obesity as a result of hypercortisolism, it was found that adiposity, in general, is more strongly associated with a flattened diurnal cortisol rhythm (FDCR). A study employing both WC and BMI as adiposity markers showed a negative correlation between immediate post-awakening free salivary cortisol and total area under the curve (AUC) [59]. A similar negative correlation with serum cortisol was also shown, where BMI elevation is 30% lower in those with high cortisol levels. Recently, it has been further determined that a 1-SD increment in WC and BMI corresponds to a respective decrease of 3.92% and 3.05% in MSC [60]. These results align with data obtained from a mathematical approach, which predicts that a more robust GC rhythm is equivalent to a higher stress tolerance, offering health advantages [16]. Despite the lack of significance and mechanistic understanding of the association between FDCR and CVD, an overall poorer health outcome, including inflammatory disorders, which can increase CVD risk, was identified [61].

All findings mentioned suggest a bidirectional influence of adiposity and cortisol. By redistributing white adipose tissue to the abdominal region and increasing appetite for energy-rich food, cortisol leads to hypertrophy and hyperplasia of adipocytes as a counter-mechanism [62, 63]. These result in low-grade chronic inflammation as adipocyte size can increase with both macrophage inflammation and leptin levels [57, 64]. Meanwhile, leptin, which has been known to have inhibitory effects on the HPA axis, can explain the chronic flattening of cortisol diurnal profile [60, 65].

## 8 DYSLIPIDAEMIA

Dyslipidaemia describes a perturbed fatty acid metabolism, which results from chronic adiposity [66]. Its characteristic lipid profile of high total triglycerides levels with a low high-density lipoprotein cholesterol (HDL-C) level has been found in 12 - 72% of CS patients as a consequence of chronic exposure to GC excess [27]. Besides, dexamethasone, which is a synthetic form of cortisol, was found to decrease HDL-C while increasing triglycerides in a dose-dependent manner [67].

Almost half of CVD patients were found to demonstrate low HDL-C, followed by hypertriglyceridemia. Both were found to be strongly associated with CVD risk factors, namely hypertension and diabetes mellitus [68]. This association with diabetes mellitus is further evidenced in a prospective cohort study conducted among T2DM patients, indicating that those with metabolic dyslipidaemia face greater risks of CVD events besides highlighting its greater significance in CVD risk compared to individual components of the lipid profile [69]. Formulated as the log ratio of triglycerides to HDL-C, the Atherogenic Index of Plasma has also been deemed as a reliable CVD risk marker [70].

By upregulating the expression of lipoprotein lipases, lipolytic activity is enhanced, thereby releasing more free fatty acids into circulation [71]. In corticosterone-fed mice, drug-induced inhibition of lipolysis showed protective effects against IGM and fat accumulation [72]. Permissive effects with insulin and adrenaline are also exerted by cortisol on lipid metabolism where only in the presence of either the rate appearance of glycerol and free fatty acids are increased [73]. Additionally, hepatic steatosis, a reflection of increased release of free fatty acids, was found to develop in 20% of CS patients and patients with high visceral fat areas with a 4-fold greater risk [71, 74, 75].

## 9 NON-METABOLIC FEATURES

Higher odds of spontaneous venous thromboembolism have been identified in CS patients (OR=17.82) compared to healthy controls. This hypercoagulability state particularly involves elevated von Willebrand factor (vWF) and factor VIII [FVIII], which are essential clotting proteins [76]. Besides, COVID-19 patients who received GC treatment were reported to have a 39% greater risk of developing venous thromboembolism [77]. Another study revealed an overall significant increased venous TE risk with exogenous use of GC in a dose-dependent manner [78]. Excessive blood loss in post-surgery settings was also determined in patients with lower cortisol levels [79].

The proposed mechanism includes activation of coagulation by shortening activated partial thromboplastin time whilst increasing vWF, Factor VIII, platelets, and thromboxane B2 [27]. Inhibition of fibrinolysis further worsens the situation, namely by promoting the expression of plasminogen activator inhibitor type 1 (PAI-1), in which GC has been found involved in its transcription in both animals and *in vitro* studies. Dexamethasone treatment was also found to induce levels of clotting factors, including FVIII and fibrinogen, corresponding with *in vitro* findings [80-82].

Recent randomisation analyses and one study also found a strong relationship between serum cortisol and CVD upon correction of metabolic parameters, as summarised in Table **1**. The odds ratio in a survey of oral GC users persisted even after adjustment for metabolic risk factors, which implies cortisol action on CVD progression, not solely through metabolic pathways [83, 84]. A dose-dependent mechanism has been proposed where CVD cumulative incidence is estimated to rise by 5.1% from non-users to users of ≥ 25 mg/day. Also, a greater association of both MSC and GC use with CHD instead of stroke risk implies its potential to predict distinct types of CVD [85-87].

## 10 TREATMENT FOR HYPERCORTISOLISM

### 10.1 Surgery

Numerous treatment options for hypercortisolism are available, but surgery remains the first-line treatment for CS patients. Depending on the tumour location, common surgical methods include transsphenoidal resection and adrenalectomy, in which the tumour is either in the pituitary or adrenal glands [88]. Post-adrenalectomy improvement of hypertension with a mean decrease of 2.7 mmHg and 9.3 mmHg for systolic and diastolic BP, respectively, has been observed in 60.1% of subclinical CS patients, whereas 51.5% showed attenuated T2DM. Overall, surgical remission of hypercortisolism can reduce CVD risk by improving metabolic parameters [89, 90].

### 10.2 Pharmacological Intervention

Medications are only opted when there is a contraindication for surgery, persistence, or recurrence of hypercortisolism [88]. These medications act on three different levels, which include, for instance, ACTH inhibitors at the pituitary level, steroidogenesis inhibitors at the adrenal level, and GR antagonists at the cellular level [90].

Pasireotide, an ACTH inhibitor, was found to be effective at lowering CVD risk by decreasing cortisol levels. A 5-year follow-up study on pasireotide treatment for Cushing’s Disease (CD) patients showed alleviation of cardiovascular-related CD manifestations [91]. Two other studies with a 1-year and 6-month follow-up showed similar results, suggesting pasireotide-induced hyperglycaemia at the same time [92, 93]. This adverse effect can be allayed by pairing pasireotide treatment with incretin-based therapy [94].

Inhibiting steroidogenesis involves interference with the process of synthesising cortisol at the adrenal level. The main target here is 11-β hydroxylase, which catalyses the final step of cortisol synthesis. One 11-β hydroxylase inhibitor that has been found effective in decreasing cortisol levels upon going through phases of clinical trials is osilodrostat [95-97]. Improvements in metabolic-related CVD risk factors, such as hypertension and IGM, have been indicated in an extensive study, followed by lowered WC [98]. Another potent 11-β hydroxylase inhibitor is metyrapone, despite showing less effectiveness than osilodrostat. Compared with osilodrostat, a less pronounced serum cortisol declination during the 12-week treatment and an antihypertensive effect in the metyrapone group have been demonstrated [99].

Mifepristone, as mentioned, exerts antihypertensive function by antagonising cortisol action and thus reduces CVD risk. Besides, improved IS and enhanced insulin-dependent glucose uptake were found to be associated [100].

Having understood the relationship between stress and cortisol, magnesium, which is closely related to stress, is indicated. Given their inverse relationship, magnesium can lower CVD risk by lowering cortisol levels [101]. A meta-analytical study has determined a 30% lower CVD risk with every 0.2 mmol/L increment of circulating magnesium. Although dietary magnesium was not found to be significantly associated with total CVD risk, it may harbour the potential to reduce CHD risk [102]. The recent findings suggest that magnesium supplementation can reduce cortisol levels by enhancing 11β-HSD2 activity, which favors the inactivation of cortisol [103].

## 11 LIMITATIONS AND CONSIDERATIONS

Despite ample evidence underlining the potential development of serum cortisol as a CVD risk biomarker, uncertainties arise whenresearchers proposed a lack of significant associations. A bidirectional analysis in 2020 showed no significant correlation between genetically predicted cortisol with CHD, ischaemic stroke, and T2DM [104]. Another study evaluating cortisol as a predictive marker in CVD showed similar results, specifically with metabolic parameters [105]. Meanwhile, a 27% lower risk of venous thromboembolism was also found to be associated with a 1-SD increment of serum cortisol, possibly mediated by high BP [106]. Nonetheless, high BP, which predisposes individuals to atrial fibrillation, as found to be positively associated with serum cortisol, should also be taken into account [56].

Another aspect is that the normalisation of circulating cortisol levels allays metabolic parameters, which are contributory to CVD risk, but an overall greater-than-normal risk persists. Irreversible vascular damage, including enhanced carotid artery stiffness and plaque formation, can be held accountable. Its potential as a risk biomarker is, therefore, impeded in those who have normal cortisol levels but with a medical history of hypercortisolism [23].

Inconsistent findings of association between cortisol levels and metabolic syndrome, mainly due to the pattern of cortisol release across a day, further impose uncertainties. FDCR, arising from dysregulation of the HPA axis, comprises both hypo- and hypercortisolism. Adverse physical health outcomes, including adiposity, BMI, inflammation and diabetes, all of which increase CVD risk, have been found to be significantly associated [61]. Moreover, a higher likelihood of coronary calcification in the quartile of flattest slopes, while showing no linear association with AUC, limits serum cortisol measurement in risk evaluation to a greater extent [107]. Odds of incident diabetes were also lower in those with more robust CAR, meanwhile to increase with a flatter late decline slope. Preclinical studies have determined its underlying pathophysiology as adiposity and hyperinsulinemia, driven by a flattening of the cortisol diurnal profile [108].

Although the negative correlation between MSC and adiposity has been well-elucidated (see *Adiposity*), contradictory results where higher MSC corresponded to increased BMI were revealed [59, 60, 109]. Adiponectin, which has been known to confer a cardioprotective effect, may instead play a paradoxical role if in excess [110, 111].

A lack of detection sensitivity for minimally elevated cortisol levels, mostly in NFAI cases, is also another limitation when considering its biomarker potential. Apart from showing no difference in metabolic parameters between autonomous cortisol-secreting adrenal incidentalomas and NFAI, NFAI poses a greater risk for incident diabetes with adjusted cortisol levels and BMI. Here, it is hypothesised that NFAT might be secreting undetectable amounts of cortisol up to within ‘normal’ limits, thus compromising its potential [112, 113].

Measuring hair cortisol concentrations (HCC) is a rising trend that could substitute serum cortisol in the future. In contrast with the short-term HPA activity reflected by serum cortisol, HCC has shown activity in the last three months. Additionally, the collection of serum samples involving venepuncture induces the stress response, from which higher cortisol levels could be falsely determined.

Apart from the usual variation shown between individuals with and without metabolic syndrome, recent studies highlighted that HCC is also significantly associated with CVD incidence, which was lacking in serum cortisol, as favoured by a meta-analysis of 26 studies [114-116]. In accordance with these studies, Andreas *et al*. even revealed a possibility of a direct association between HCC and CVD, independent of metabolic features [117]. This consolidates findings for an increased risk of CVD following serum cortisol level measurement upon correction of metabolic parameters. This suggests that HCC presents the same niche as serum cortisol but with potentially enhanced consistency. Notably, various methods, including chemiluminescent immunoassay and liquid chromatography-mass spectrophotometry, have been established for the measurement of HCC, which further consolidates its practicality in routine diagnostic settings [118, 119].

## CONCLUSION

In conclusion, ample studies have demonstrated a strong evidence-based relationship between CVD risk and cortisol levels. In addition to this, their significant association would be through well-identified CVD risk factors, especially in terms of metabolic features. Nonetheless, the potential of serum cortisol in predicting CVD risk has been limited due to inconsistent findings on their correlation. Specifically, it was unclear whether higher levels of the hormone, flattening of the cortisol curve, or both are involved. Findings that highlight a potential direct association between cortisol and CVD also raise questions about its underlying pathogenesis, if not through metabolic mechanisms. Therefore, further research should explore the prospect of serum cortisol either as a risk or prognostic marker for CVD, as well as elucidate the role of cortisol diurnal profiles in modulating disease progression and their correlation with CVD.

## Figures and Tables

**Table 1:** Odds ratio (OR) for CVD/Stroke following the 1-SD increase in serum cortisol.

**-**	**-**	**No. of Participants**	**OR**	**References**
Randomisation analyses	The northern sweden VIP, MONICA and MSP study	2622	1.31	[86]
	British women’s heart and health study	4286	1.13	
	Caerphilly study	2512	1.10	
	Vietnam experience study	4255	1.40	
Retrospective cohort study	Serum cortisol, 25 [OH]D, and cardiovascular risk factors in patients with type 2 diabetes mellitus	762	1.25	[87]

**Note:** OR = odds ratio.

## LIST OF ABBREVIATIONS

11β-HSD: 11β-Hydroxysteroid Dehydrogenase
11β-HSD1: 11β-Hydroxysteroid Dehydrogenase Type 1
11β-HSD2: 11β-Hydroxysteroid Dehydrogenase Type 2
ACTH: Adrenocorticotropic Hormone
AUC: Area Under Curve
BMI: Body Mass Index
BP: Blood Pressure
CAR: Cortisol Awakening Response
CHD: Coronary Heart Disease
CS: Cushing’s Syndrome
CVD: Cardiovascular Disease
DST: Dexamethasone Suppression Test
FDCR: Flattened Diurnal Cortisol Rhythm
FVIII: Factor VIII
GC: Glucocorticoid
GR: Glucocorticoid Receptor
HCC: Hair Cortisol Concentrations
HDL-C: High-density Lipoprotein Cholesterol
HPA: Hypothalamic-pituitary-adrenal
IR: Insulin Resistance
IGM: Impaired Glucose Metabolism
MR: Mineralocorticoid Receptor
MSC: Morning Serum Cortisol
NFAI: Non-functioning Adrenal Incidentaloma
SD: Standard Deviation
T2DM: Type 2 Diabetes Mellitus
vWF: Von Willebrand Factor
WC: Waist Circumference
